# Evaluation of the clinical usefulness of pancreatic alpha amylase as a novel biomarker in dogs with acute pancreatitis: a pilot study

**DOI:** 10.1080/01652176.2024.2326007

**Published:** 2024-03-18

**Authors:** Keon Kim, Hee-hong Kim, Jae-Beom Joo, Ock-Kyu Kim, Sin-Wook Park, Guk-Hyun Suh, Woong-Bin Ro, Chang-Min Lee

**Affiliations:** a Department of Veterinary Internal Medicine, College of Veterinary Medicine and BK21 FOUR program, Chonnam National University, Gwangju, Korea; b Cool-pet Animal Hospital, Daegu, Korea

**Keywords:** Acute pancreatitis, dogs, pancreatic-alpha amylase

## Abstract

Pancreatic alpha amylase (P-AMY) is used as a biomarker of acute pancreatitis (AP) in human medicine. To our knowledge, there are no studies evaluating the usefulness of P-AMY in dogs with AP. In this study, we evaluated the diagnostic value of P-AMY, currently not verified in veterinary medicine. The AP group (*n* = 40) consisted of dogs with AP diagnosed using clinical signs and laboratory examinations, including abnormal canine pancreatic lipase (cPL) concentration, and compatible abdominal ultrasound examination at first presentation. Evaluation of the canine AP severity (CAPS) score was performed. The control group (*n* = 38) was composed of normal dogs without any abnormalities in clinical findings, blood exams or diagnostic imaging. The correlation of P-AMY with cPL was confirmed by Pearson’s correlation analysis (*r* = 0.564, *p* < .001). The sensitivity and specificity for the most appropriate cut-off values of P-AMY were recorded similar to the values of DGGR. The dogs with AP and CAPS ≥11 had significantly higher serum P-AMY (*p* = .016) contrary to DGGR lipase and cPL. Furthermore, there was a significant difference in the median P-AMY dependent on the presence of systemic inflammatory response syndrome (*p* = .001). P-AMY showed similar level of diagnostic accuracy along with sensitivity and specificity compared to DGGR lipase. In addition, P-AMY showed a significant association with CAPS score, contrary to cPL and DGGR lipase. Along with other biomarkers associated with AP, P-AMY has the potential of usefulness as a supportive diagnostic and prognostic biomarker of AP in dogs.

## Introduction

Acute pancreatitis (AP) is an exocrine pancreatic disease frequently encountered in dogs (Watson, 2015). Although histopathological examination is the definitive diagnostic method of pancreatitis, it has limitations (Kim et al., 2017). Therefore, non-invasive diagnostic procedures such as the measurement of serum canine pancreatic-specific lipase (cPL) and ultrasonographic evaluation of the pancreas, have been generally considered as the reference standard for diagnosis of canine pancreatitis in clinical practice (Xenoulis, 2015).

The cPL immunoreactivity assay is routinely used to diagnostic laboratory test for canine pancreatitis (McCord et al., 2012). However, false positive and false negative results have been reported. For instance, exogenous and endogenous glucocorticoids affect serum cPL concentration (Mawby et al. 2014). Moreover, dogs with upper gastrointestinal obstruction showed an increase in pancreas specific lipase into the circulation, without significant pancreatic disease (Lidbury and Suchodolski 2016).

Recently, the activity of 1,2-o-dilauryl-rac-glycero-3-glutaric acid-(6′-methylresorufin) ester (DGGR) is considered to have validity (Graca et al. 2005). Correlation between DGGR lipase activity and cPL concentration is strong and showed almost identical results based on several reports (Kook et al. 2014; Cridge et al. 2018; Goodband et al. 2018; Wolfer et al. 2022; Cuneni et al. 2023; Hammes and Kook 2022). However, as with cPL application, the diagnostic performance of DGGR lipase is dependent on the type of study and the cutoff value used (Hope et al. 2021).

Alpha-amylase (AMY) is an enzyme produced in mammals including dogs and preserved primarily in salivary glands and the pancreas, represented by saliva (S) and pancreas (P). Total AMY is derived from various organs, so an increase in the level of total AMY does not necessarily indicate pancreatic disorders. However, in human medicine, direct measurement of pancreatic isoenzyme of amylase (P-AMY) based on selective isoenzyme inhibition by monoclonal antibodies is possible. In addition, P-AMY displays prominent precision, reliability, and practicability in the diagnosis of acute pancreatitis (Italian Society of Clinical Biochemistry and Clinical Molecular Biology \(SIBioC\) Working Group on Enzymes 2002) and has utility as serological biomarker (Italian Society of Clinical Biochemistry and Clinical Molecular Biology \(SIBioC\) Working Group on Enzymes 2002; Choi et al. 2003).

To the best of our knowledge, there have been no studies evaluating the usefulness of P-AMY as a novel biomarker in veterinary medicine. In this study, usefulness of P-AMY was confirmed in canine AP for the first time. Furthermore, we investigated its advantages as a novel biomarker by comparing it with other serological biomarkers in canine AP.

## Materials and methods

### (1) Serum sampling

Serum P-AMY concentrations were measured using surplus serum from hospitalized dogs with AP at our veterinary teaching hospital. All blood samples were obtained at the time of presentation and before any treatment was performed. Serum was acquired by centrifugation at 10,000 g × 3 min within 1 hour, and analysis of serum enzymes was performed immediately afterwards. The experimental design was approved by the University Institutional Animal Care and Use Committee (Approval number: CNU IACUC-YB-2022-61).

### (2) Animals

The AP group (*N* = 40) was selected retrospectively among dogs who were diagnosed with acute pancreatitis at the first presentation in our veterinary teaching hospital. Dogs received any treatment within 7 days before the presentation were excluded in AP group. The diagnostic criteria of AP were determined as follows: apparent clinical signs within seven days prior to the first presentation (more than two events of abdominal pain, diarrhea, vomiting, decreased appetite and/or lethargy), changes of shape and echogenicity consistent with AP on abdominal ultrasound examination (Prosound a7, Hitachi Aloka Medical, Ltd., Japan) at the first presentation and abnormal result of a cPL kit test (Canine SNAP cPL^TM^; IDEXX Laboratories Inc., USA). All ultrasound examinations were conducted by an experienced veterinary radiologist and were considered as AP on diagnostic imaging if enlargement of hypoechoic pancreas was detected along with irregular shape and margins, and hyperechoic mesenteric fat near the pancreas.

All control group (*N* = 38) animals consisted of healthy dogs with no pancreatic disease, or systemic inflammation nor neoplasia based on history, physical examination, blood test results (complete blood count, serum biochemistry) and diagnostic imaging (radiography, abdominal ultrasound). Furthermore, all dogs having any possibility of exposure to endogenous (e.g. hyperadrenocorticism) or exogenous glucocorticoids were excluded.

### (3) Laboratory examinations

The P-AMY assay was performed using an auto chemistry colorimetric analyzer (Dotto 2000 Auto Chemistry Analyzer, MTD Diagnostics Co., Italy) with pancreatic α amylase reagent (LC Diagnostics Co., South Korea). The measurement of DGGR lipase activity by enzymatic analyzer (Eurolyzer Solo, Eurolyzer Diagnostica GmbH Co., USA; Reference interval [RI], 0–125 U/L) was performed for dogs tentatively diagnosed with AP by the results of a SNAP cPL kit and ultrasound evaluation. Of note, cPL was quantified using a multi-purpose quantitative automatic in vitro diagnostic device (Vet Chroma™, ANIVET, South Korea; RI, 0–200 ng/mL), reported to has the 94.44% sensitivity and 100% specificity based on laboratory cPL immunoreactivity (Spec cPL®, IDEXX Laboratories, USA) (Kang 2020). A serum chemistry analyzer (Catalyst Dx Chemistry Analyzer, IDEXX Veterinary Diagnostics Co., USA) was used for quantifying other serological enzymes such as creatinine and c-reactive protein. Hematologic parameters were measured using a complete blood cell count analyzer (Procyte Dx analyzer, IDEXX Veterinary Diagnostics Co., USA). Parameters of secondary hemostasis (prothrombin [PT], activated partial thromboplastin time [aPTT]) were evaluated by using automatic hematology coagulation analyzers (CG02NV, A&T Co., Japan). Ionized calcium concentration was quantified by using a blood gas and electrolytes analyzer (pHOx Ultra, Nova Biomedical Co., South Korea).

### (4) Canine acute pancreatitis severity score

Canine acute pancreatitis severity (CAPS) score, a recently published clinical scoring system for predictors of early death in dogs with AP (Fabrès et al. 2019), was evaluated for all dogs at the time of presentation. The CAPS score was calculated as follows: CAPS score = 8 × (1 if systemic inflammatory response syndrome [SIRS], 0 otherwise) + 3 × (1 if coagulation disorders, 0 otherwise) + 4 × (1 if increased serum creatinine concentration, 0 otherwise) + 3 × (1 if ionized hypocalcemia, 0 otherwise). The presence of SIRS was evaluated by using the proposed criteria. A dog was classified in the SIRS group if at least two of the following four criteria were met: hyperthermia or hypothermia (>39.7 or <37.8 °C), tachycardia (>160 beats/min), tachypnea (> 40 breaths/min), white blood cell count < 4000/μL or >12 000/μL or band neutrophils >10%. Dogs with coagulation disorders were assigned upon the presence of at least one of the following criteria: thrombocytopenia (platelet count <148 000/uL), prothrombin time, activated partial thromboplastin time prolonged by more than 25% of the upper end of the reference interval.

### (5) Statistical analyses

All statistical data were analyzed using commercially available software (IBM SPSS Statistics, version 26, IBM Co., USA and GraphPad Prism v9.0, GraphPad Software Inc., USA). The Shapiro-Wilk test was performed to determine normality. Parametric student’s *t* test was performed to confirm the significance of various biomarkers between the AP group and the control group. Non-parametric Mann-Whitney *U* test was conducted to compare AP biomarkers between two groups, subdivided by the CAPS cutoff. Pearson’s correlation analysis was used to assess whether an association of cPL with DGGR lipase and P-AMY. Spearman’s correlation analysis was conducted to confirm the association between P-AMY and other inflammatory parameters in AP dogs with previous measured data (non-parametric). Receiver operating characteristic (ROC) curves were used to compare the diagnostic sensitivity and specificity of P-AMY with DGGR lipase. Statistical significance was set *p* < .05 for all analyses.

## Results

### (1) Study population

Forty dogs diagnosed with AP were enrolled in this study. Mean age was 10.1 ± 4.4 years. The most common breeds were Poodle (*n* = 9), Maltese (*n* = 6), Yorkshire Terrier (*n* = 5), Pomeranian (*n* = 4), American Pit Bull Terrier (*n* = 3), Miniature Schnauzer (*n* = 2), Shih Tzu (*n* = 2), Old English Bulldog (*n* = 2) and the other seven dogs (17.5%) were mixed breeds. Of the dogs with AP (*n* = 40), 22 animals were female (55%, 14 were spayed) and the remaining 18 were male (45%; 15 were castrated). The mean body weight was 6.4 ± 5.9 kg, ranging from 1.82 to 23.9 kg. The CAPS score was evaluated for 31 dogs that could be scored based on chart data and blood examination in 40 dogs with AP. In 31 of 40 dogs, the CAPS score ranged from 0 to 15 with a median of 4. Three dogs had CAPS scores of 3, five dogs had CAPS scores of 4, three dogs had CAPS scores of 7 and three dogs had CAP scores of 8. Meanwhile, five dogs and one dog had CAPS scores of 11 and 15, respectively. The CAPS scores of other dogs (*n* = 11) were calculated as 0. The control group (*n* = 38) consisted of Beagle (*n* = 24), Maltese (*n* = 5), Poodle (*n* = 2), Dachshund (*n* = 1), Shih-Tzu (*n* = 1), Korean Jindo (*n* = 1) and four mixed-breed dogs 7.2 ± 5.5 years of age. Mean weight of the control group was 7.1 ± 4.9 kg.

### (2) Activity of serum P-AMY, DGGR lipase and cPL in control dogs and dogs with AP

**Figure 1.**
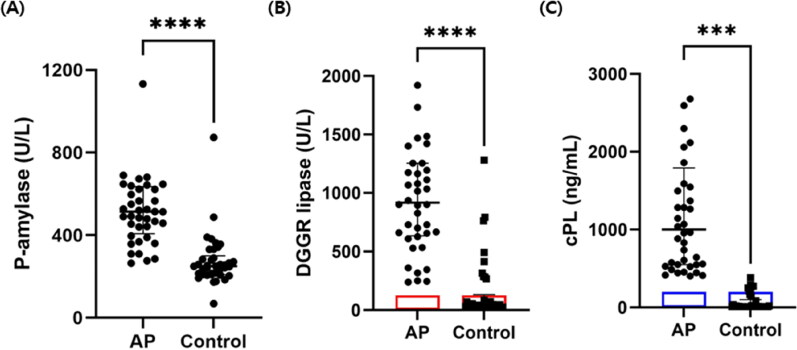
Scatter dot plot comparing median P-amylase, DGGR lipase and cPL concentration between control dogs (*n* = 38) and dogs with AP (*n* = 40). All enzymes in dogs with AP were significantly higher than control group (student’s t test; *p* < .001 in two enzymes [P-amylase, DGGR lipase] and *p* = .001 in cPL). Two, three and four data points in AP are outside the axis limits for visualization in figure (a), (B) and (C), respectively. The red box is the RI of DGGR lipase (0–125 U/L), and the blue box showed the RI of cPL (0–200 ng/mL). ***p <.001, ****p <.0001.

The dogs with AP had significantly higher median serum P-AMY compared to the control group (median, 502.2 U/L; interquartile range [IQR], 406.9–622.1 versus median, 249.6 U/L; IQR, 209.3–299.7; *p* < .001; Figure 1(A)). Likewise, the dogs with AP exhibited significantly higher median serum DGGR lipase concentration than the control group (median, 918.3 U/L; IQR, 643.7–1254 versus median, 43.8 U/L; IQR, 28.48–131.7; *p* < .001; Figure 1(B)). The concentration of cPL also showed a similar pattern in the AP group as compared to the control group (median, 1002 ng/mL; IQR, 550.2–1790 versus median, 10.35 ng/mL; IQR, 10.0–101.3; *p* < .001; Figure 1(C)). All described data of serum P-AMY, DGGR lipase and cPL are visualized in Table 1.

**Table 1.** Serological activity of the enzymes in control dogs and dogs with acute pancreatitis.

Group	P-amylase (U/L)	DGGR lipase (U/L)	cPL (ng/mL)
	Median (quartiles)	Median (quartiles)	Median (quartiles)
Controls (*n* = 38)	249.6 (209.3–299.7)	43.8 (28.48–131.7)	10.35 (10.0–101.3)
Acute pancreatitis (*n* = 40)	502.2 (406.9–622.1)	918.3 (643.7–1254)	1002 (550.2–1790)

### (3) Correlation of serum cPL with DGGR lipase and P-AMY results

**Figure 2.**
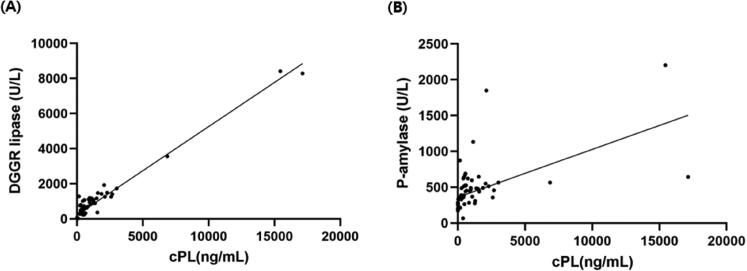
Scatterplot of canine pancreatic lipase (cPL) concentration with DGGR-lipase (A) and P-AMY (B). Pearson’s correlation analysis displayed with significant correlation (*p* < .001) between cPL and DGGR-lipase as well as P-AMY (pearson’s correlation coefficient (*r*) = 0.979 and 0.564; a linear regression equation: y = 0.5x + 226 and 0.067x + 363, respectively).

The correlation between cPL and DGGR lipase showed a Pearson’s correlation coefficient of *r* = 0.979 (*p* < .001) and a linear regression equation y = 0.5x + 226 (Figure 2(A)). The correlation of cPL with P-AMY displayed a Pearson’s correlation coefficient of *r* = 0.564 (*p* < .001) and a linear regression equation y = 0.067x + 363 (Figure 2(B)).

### (4) Evaluation of sensitivity and specificity

In this study, the most appropriate cut-off values obtained from the ROC curves were 356.3 U/L for P-AMY and 316.5 U/L for DGGR lipase. The specificity of the most appropriate cut-off values was 87.5% for P-amylase and 92.5% for DGGR lipase, and sensitivity was identical at 86.84% (Table 2).

**Table 2.** Clinical sensitivities and specificities of P-amylase and DGGR lipase.

	Best cutoff value	
	P-amylase (356.3 U/L)	DGGR lipase (316.5 U/L)
Sensitivity (%)	86.84%	86.84%
Specificity (%)	87.5%	92.5%

### (5) Comparison of diagnostic accuracy through the receiver operating characteristic curve analysis

**Figure 3.**
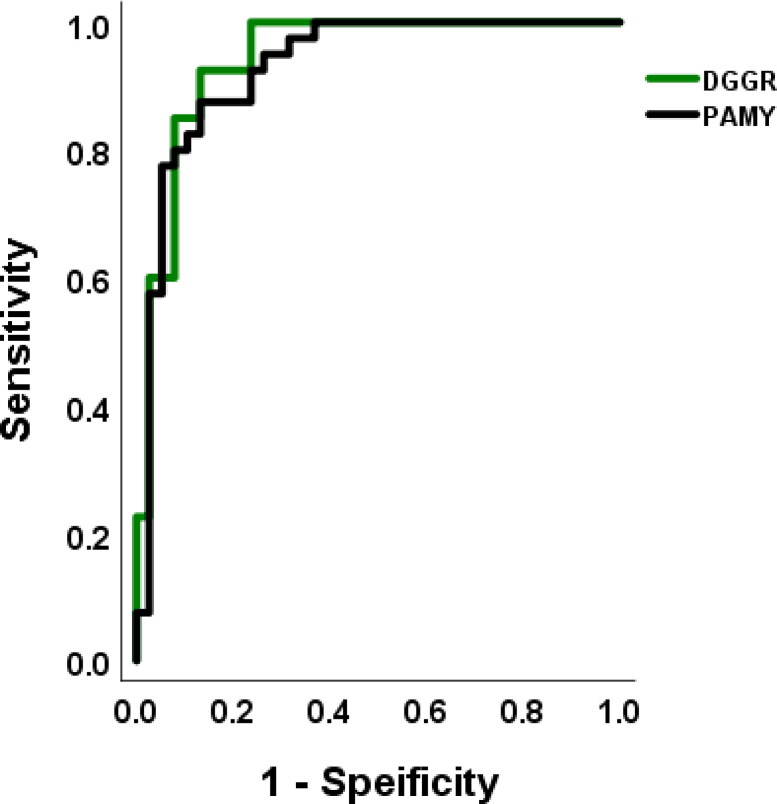
Receiver operator characteristic curves of DGGR lipase and P-AMY of acute pancreatitis. The area under the curves were estimated as 0.930 for P-AMY and 0.943 for DGGR-lipase.

In the analysis of the ROC curves, the area under the curve (AUC) was confirmed as 0.930 for P-AMY and 0.943 for DGGR lipase (Figure 3). As cPL is used as the serological gold standard for the diagnosis of AP, the sensitivity and specificity of cPL were not evaluated. Of note, all dogs with AP showed values of ≥ 400 ng/mL.

### (6) Serum P-AMY concentration between dogs with AP and control dogs and the association between P-AMY and CAPS score

**Figure 4.**
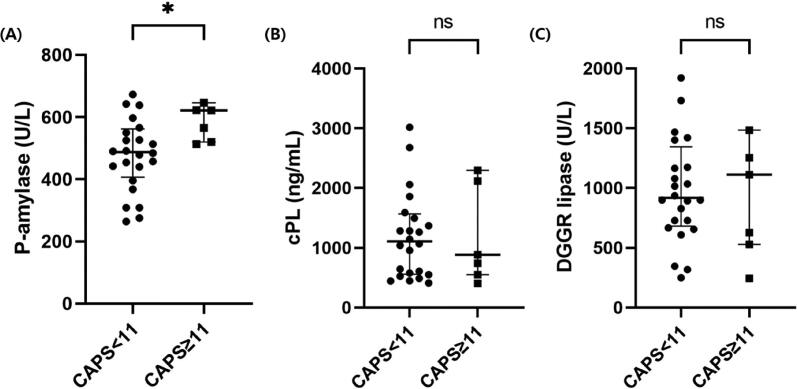
Scatter dot plot comparing serum P-AMY, DGGR lipase and cPL concentrations between two groups, which were subdivided using the validated CAPS cutoff (CAPS ≥11 and <11). Dogs with CAPS ≥11 (*n* = 7) showed a significantly higher median P-AMY concentration than AP dogs with CAPS <11 (*n* = 24) (Mann-Whitney test, *p* = .016). In contrast, DGGR lipase and cPL concentration did not have significant difference with CAPS score (*p* = .975 and *p* = .865, respectively). Two data points are outside the axis limits to visualize the comparison between groups (CAPS < 11 and CAPS ≥11) in figure (a), (B) and (C). *p < .05, ns: no statistical significance between groups.

Among the 40 dogs with AP, 31 dogs whose CAPS score could be calculated were selected. Those AP dogs with CAPS ≥11 (*n* = 7) had a significantly higher serum P-AMY than did dogs with CAPS <11 (*n* = 24) (621.7 U/L; IQR, 519.9–646.0 vs 487.1 U/L; IQR, 406.9–561.8; *p* = .016; Figure 4(A)). For DGGR lipase and cPL, there were no significant difference between AP dogs with CAPS ≥11 and AP dogs with CAPS <11 (*p* = .975 and *p* = .865, respectively; Figure 4(B and C)). SIRS was present in nine dogs (29.03%). A significant difference of median serum P-AMY was observed between dogs with and without SIRS (596.87 vs 487.08 U/L; *p* = .001). However, there were no significant correlations between P-AMY and other inflammatory parameters such as CRP and WBC (*p* = .250 and .594, respectively).

## Discussion

P-AMY, a pancreatic-specific amylase, is used as a biomarker of AP in human medicine (Ismail and Bhayana 2017). As it is now possible to measure P-AMY, the sensitivity and specificity of diagnosing AP have advanced (Moridani and Bromberg 2003; Yang et al. 2005). However, measurement of pancreatic amylase has been largely ignored due to the superiority of lipase-only measurements in diagnosing AP, an increase in the expense of measuring pancreatic lipase, and the discomfort of various equipment platforms (Tietz et al. 1988; Yadav et al. 2002, 2011). Therefore, limited reports on P-AMY were found in human studies and total amylase has been mostly used for the diagnostic purposes of AP until now. Furthermore, there have been few reports on the use of pancreatic amylase for diagnostic purposes in canine AP. The authors reviewed 20 research articles published between 1970 and 2023 that measured serum amylase in dogs with AP. Among the 20 papers, 19 did not clearly specify whether the source of serum amylase activity was derived from pancreas or referred to total amylase. Although the source was not specified, these studies may be presumed to have measured serum total amylase. Only one paper was found to measure both total amylase and P-AMY, which study was performed in canine models with experimentally-induced AP (Petrova 2017). In this study, we evaluated the diagnostic usefulness of P-AMY in AP, for the first time in veterinary medicine. P-AMY has the potential as supportive biomarkers for diagnosis and prognosis of AP utilized with other diagnostic parameters associated with AP in the field of small animal veterinary clinical medicine.

According to a recent study, DGGR lipase could originate not only from the pancreas but also from lipoproteins and the liver (Lim et al. 2020). However, we had difficulty in measuring lipoprotein directly and identifying hyperlipidemia, due to the retrospective nature of the study. As the measurement of P-AMY is only quantified by pancreas-derived enzyme, it is thought that the best cutoff value of P-AMY is less prone to external factors, contrary to DGGR lipase. Considering that the sensitivity and specificity of P-AMY show a similar level as compared to DGGR lipase, measuring the P-AMY along with DGGR lipase could be useful in making a more accurate diagnosis of AP.

To decrease the death rate from severe AP, early identification is essential to initiate proper treatment as early as possible (American College of Gastroenterology 2013). The death rate in dogs with AP remains high (27% to 58%) although the knowledge about AP is considerable (Cook et al. 1993). As a result, providing proper treatment and reducing death rates, the severity assessment of AP in dogs should be established (Cook et al. 1993; Mansfield et al. 2003). The CAPS score is a validated clinical scoring system developed recently for short-term mortality in dogs with AP (Fabrès et al. 2019). Using the CAPS score, other biomarkers have been evaluated for the prognosis of AP (Gori et al. 2020). In this study, we calculated the CAPS score for 31 of 40 cases with AP at the time of presentation. Following the previous study, the best cut-off values for optimal diagnosis were set as CAPS ≥11 (Fabrès et al. 2019). As for P-AMY, it displayed significantly high values in CAPS ≥11 dogs with AP as compared to CAPS <11 AP dogs. This finding was contrary to cPL and DGGR lipase which did not display any significant differences. To date, blood tests for discriminating the severity of disease do not exist for dogs with AP (Mansfield et al. 2003; Ruaux and Atwell 1998). We confirmed that P-AMY is helpful in evaluating the severity of AP using only one blood measurement in small animal clinical medicine.

In human medicine, there is an association between the presence of SIRS and death in severe AP cases within the first 48 h (American College of Gastroenterology 2013; Mofidi et al. 2006). In addition to CAPS score evaluation, the presence of SIRS and the results of complete blood cell count and serum chemistry related to inflammation were analyzed. There was a significant difference in the median of P-AMY concentrations dependent on the presence of SIRS (*p* = .001). This indicates a high correlation between the P-AMY concentration and SIRS, which can occur as a complication of AP. In addition, it is consistent with the fact that SIRS is the manifestation with the highest weight of the CAPS score, related to the severity of AP. In contrast, systemic inflammatory biomarkers and indicators such as CRP and WBC did not show any significant correlations with P-AMY, meaning that P-AMY is not related to amounts of inflammatory biomarkers in blood. The diagnostic criteria for SIRS consider the patient’s vital signs as well as the results of inflammatory levels through examination of blood parameters (Brady et al. 2000; Greiner et al. 2008). As for P-AMY, it has the potential as a serological biomarker of pancreatitis-related prognostic evaluation in the sense that it showed a correlation with SIRS, which considers not only inflammatory values but also a patient’s vital signs.

Due to the characteristics of a retrospective study, the results of blood examination associated with inflammation could not be acquired for all cases. Second, the fact that the CAPS score could not be evaluated in all dogs with AP is considered as a study limitation. Lastly, because most cases were difficult to follow-up, it was impossible to identify survivors or decedents *via* survival analysis. To verify the advantages of P-AMY measurements, a prospective study based on a large number of cases is necessary in the future.

In this study, P-AMY is displayed with diagnostic accuracy through ROC analysis similar to DGGR lipase. Furthermore, in the CAPS scores, P-AMY exhibited significant differences among other indicators related to AP. We conclude that if P-AMY is measured with other AP diagnostic biomarkers such as DGGR lipase or cPL, it will be useful as a supportive diagnostic biomarker of AP in dogs. Additionally, P-AMY may have a possibility of indirectly predicting a patient’s prognosis.
